# Recombinant and Chimeric Disintegrins in Preclinical Research

**DOI:** 10.3390/toxins10080321

**Published:** 2018-08-07

**Authors:** Victor David, Barbara Barbosa Succar, João Alfredo de Moraes, Roberta Ferreira Gomes Saldanha-Gama, Christina Barja-Fidalgo, Russolina Benedeta Zingali

**Affiliations:** 1 Laboratório de Hemostase e Venenos, Instituto de Bioquímica Médica Leopoldo de Meis, Universidade Federal do Rio de Janeiro, Rio de Janeiro 21.941-902, Brazil; victorvdavid@gmail.com (V.D.); bbsuccar@gmail.com (B.B.S.); 2 Instituto de Ciências Biomédicas, Universidade Federal do Rio de Janeiro, Rio de Janeiro 21.941-902, Brazil; joaomoraes@icb.ufrj.br; 3 Laboratório de Farmacologia Celular e Molecular, Departamento de Biologia Celular, IBRAG, Universidade do Estado do Rio de Janeiro, Rio de Janeiro 20.551-030, Brazil; saldanhagama@yahoo.com.br (R.F.G.S.-G.); barja-fidalgo@uerj.br (C.B.-F.)

**Keywords:** Snake venom disintegrin, integrin, cancer

## Abstract

Disintegrins are a family of small cysteine-rich peptides, found in a wide variety of snake venoms of different phylogenetic origin. These peptides selectively bind to integrins, which are heterodimeric adhesion receptors that play a fundamental role in the regulation of many physiological and pathological processes, such as hemostasis and tumor metastasis. Most disintegrins interact with integrins through the RGD (Arg-Gly-Asp) sequence loop, resulting in an active site that modulates the integrin activity. Some variations in the tripeptide sequence and the variability in its neighborhood result in a different specificity or affinity toward integrin receptors from platelets, tumor cells or neutrophils. Recombinant forms of these proteins are obtained mainly through *Escherichia coli*, which is the most common host used for heterologous expression. Advances in the study of the structure-activity relationship and importance of some regions of the molecule, especially the hairpin loop and the C-terminus, rely on approaches such as site-directed mutagenesis and the design and expression of chimeric peptides. This review provides highlights of the biological relevance and contribution of recombinant disintegrins to the understanding of their binding specificity, biological activities and therapeutic potential. The biological and pharmacological relevance on the newest discoveries about this family of integrin-binding proteins are discussed.

## 1. Introduction

Snake venoms contain several components, some of which have given rise to drugs and diagnostic tools for diseases [1]. Among these elements the disintegrins, a family of small peptides (4–16 kDa) rich in cysteine, deserve our attention. Disintegrins are found in the venoms of *Atractaspididae*, *Colubridae*, *Elapidae*, and *Viperidae* snake families. They selectively bind to integrin receptors [2,3], which are glycoproteins that play a fundamental role in the regulation of many physiological and pathological processes, such as hemostasis [4], thrombosis, cell adhesion, angiogenesis, proliferation [5] and tumor metastasis [6]. Most of the disintegrins interact with integrins through the RGD (Arg-Gly-Asp) sequence loop [7,8].

Previous reviews on disintegrins reported overviews of applications of these peptides in cancer research, platelet aggregation, endothelial cells and other biological processes [2,3,9,10,11,12,13,14,15,16]. Here we focus exclusively on the biological relevance and insights provided by the use of recombinant disintegrins. These recombinants proteins have been particularly valuable for the comprehension of biological activities, structure and the potential for the use of disintegrins as drugs.

### 1.1. Historical Overview

Discoveries related to disintegrins began to appear in the 1980s with a study by Huang et al. [17]. The first isolated disintegrin, trigramin, was obtained from the crude venom of *Trimeresurus gramineus* (*Viperidae family*). It was shown to be a small non-enzymatic protein that was capable of inhibiting the platelet aggregation induced by ADP (adenosine diphosphate) [17]. The term disintegrin was used for the first time in 1990, with the proposal to designate a group of peptides from the snake venoms of *Viperidae*—specifically, those with the ability to inhibit platelet aggregation through the platelet integrin αIIbβ3 [2].

These finds opened a field for many researchers in the journey to understand the interaction between disintegrins and the related receptors, integrins. Some of these low-molecular-weight peptides have led to the development of new drugs such as Integrillin [1].

### 1.2. Integrins on Platelets, Tumors and Leucocytes

Integrins can be described as heterodimeric receptors on cell surfaces that mediate cell adhesion. Many cellular processes are related to these receptors, including hemostasis, cell survival, migration, proliferation, angiogenesis, and inflammatory activity [4,5,18]. In addition to major roles in physiological processes, integrins have been implicated in pathological angiogenesis and metastasis, where they become important pharmacological targets [19].The integrin αIIbβ3, also known as glycoprotein GPIIb/IIIa, plays an important role in the process of platelet aggregation and is the most abundant receptor on the platelet’s surface. One of the essential steps in this process is the link between integrin αIIbβ3 and fibrinogen through the RGD motif. Since this receptor is also involved in pathological conditions such as acute coronary ischemia and thrombosis, integrin αIIbβ3 is the main target receptor on platelets during thrombotic events [20,21]. 

Integrins αvβ3 and α5β1 are implicated in tumor growth and tumor angiogenesis. Angiogenesis is an important adjunct to tumor growth, because the development of new blood vessels can supply oxygen and nutrients to clusters of tumor cells, contributing to tumor development [22]. The integrin αvβ3 mediates cell adhesion, an important step for survival of many types of cells [23]. This heterodimeric receptor can bind to different components of the extracellular matrix (ECM), among them vitronectin and fibronectin [24], and induce migration in endothelial cells [25] and tumor cells [26]. The integrin α5β1 interacts with fibronectin, contributing to migration and proliferation of tumor cells and other components of the ECM [22].

Some integrins not only promote angiogenesis and tumor growth, but also share in important immune processes, including inflammatory response and leukocyte trafficking. This group includes the integrins αMβ2 and α4β1, capable of mediating leukocyte-endothelial interactions [27]. 

### 1.3. Structural Classification of Snake Venom Disintegrins

Disintegrins from snake venoms originate mainly as domains of the so-called P-II snake venom metalloproteinases (SVMP) containing a disintegrin domain, which undergo proteolytic processing to generate the disintegrin. In contrast, P-III SVMP (containing disintegrin-like or cysteine-rich domains) are released as a multi-domain protein that eventually can be processed to generate fragments that correspond to a disintegrin-like/cysteine rich-like portion of the molecule [14,28]. 

**Figure 1:**
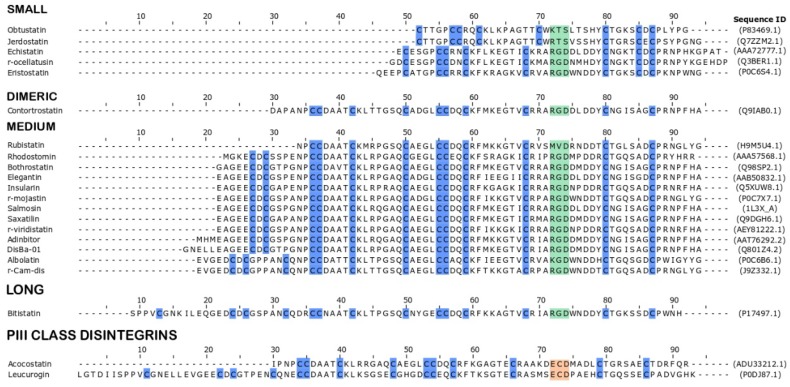
Structural classification and alignment of multiple sequences of representative disintegrins. Disintegrins are aligned by activity motif. The amino acids are represented by one-letter code. Motifs of different PII disintegrins are shown in green and the motif of PIII class disintegrins is shown in beige. Cysteine residues are highlighted in blue.

Disintegrins are classified according to number of amino acids and also number of disulfide bonds, and can be monomeric or dimeric. Three classification groups include: small peptides (41–51 amino-acid residues and four disulfide bonds), medium peptides (70 amino-acid residues and six disulfide bonds) and long peptides (about 84 amino-acid residues and seven disulfide bonds). Dimeric disintegrins consist of subunits containing about 67 amino-acid residues and ten cysteines, forming four intrachain disulfide bonds and two interchain bonds [29]. Representative members of those groups are summarized in Figure 1.

The biological activity of disintegrins is dependent on suitable cysteine pairing, which has a fundamental role related to conformation and loop mobility that is essential to the integrin-inhibitory activity [29].

### 1.4. Functional Classification of Snake Venom Disintegrins

**Figure 2:**
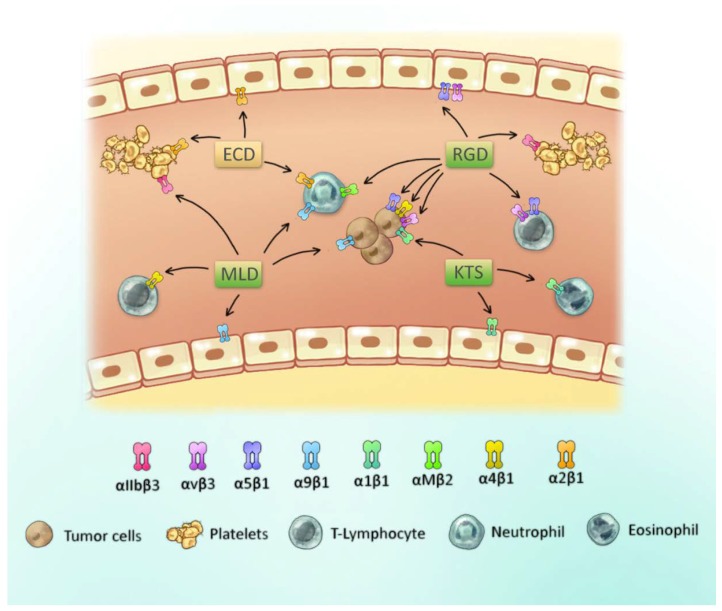
Scheme of the active site of disintegrins from snake venom and their respective binding integrin cell types. Some integrins and cell types are shown, with a focus on platelets, tumor cells and leukocytes. Disintegrin motifs are depicted in green and the PIII class disintegrin motif in beige.

Disintegrins can be classified into three different groups according to the tripeptide domain: RGD, MLD and R/KTS [14]. A scheme of these groups and their integrin-binding specificity are presented in Figure 2. The first group of disintegrins, containing the tripeptide RGD, are widely investigated. This motif results in the active site that modulates the activity of RGD-dependent integrins, such as αIIbβ3, αvβ3, α5β1, and leukocyte integrins αMβ2 and α4β1, among others [17,30]. Some disintegrins may possess a different, but related, tripeptide sequence, generally with the aspartic acid conserved, that includes the motifs MVD, KGD and WGD, among others [14]. 

This broad spectrum of integrin activity on RGD disintegrins occurs due to the presence in the protein sequence and structure of different elements that affect the biological activity of these proteins [31]. An extended conformation of the RGD motif leads to selective binding to αIIbβ3, while a bent conformation can modulate this selection to different integrins, such as αvβ3 [32,33,34]. Studies with synthetic peptides that mimic the RGD loop are reported to be less efficient than the whole molecule to target integrin αIIbβ3. Despite the lower efficiency for targeting integrins αIIbβ3, these peptides bound to integrins αvβ3, αvβ5 and α5β1 efficiently [35]. Such differences in binding affinity have been attributed to the amino-acid residues flanking the motif. Studies that exemplify this phenomenon reported disintegrins with the RGDN sequence showing better interaction with αvβ3 and α5β1 than disintegrins with the RGDW sequence. Among the studies that support these data [36,37], the one by Lucena et al. [37] evaluates the ability of disintegrins r-mojastin and r-viridistatin to bind to integrins αvβ3 and αvβ5. R-viridistatin, an RGDNP-motif disintegrin, showed a greater binding affinity for αvβ3 and αvβ5 than r-mojastin, an RGDW-motif disintegrin. This suggests that amino acids adjacent to the interaction motif can affect the selectivity of disintegrins for integrin receptors [8].

The second group of disintegrins contains the MLD motif, which is a feature of heterodimeric disintegrins that can bind to leukocyte integrins such as α4β7, α9β1 and α4β1 [38]. Some heterodimeric disintegrins may contain an RGD sequence that directs the selectivity to other integrins, such as platelet integrin αIIbβ3, among others [39,40]. 

The third group of disintegrins is composed of KTS or RTS disintegrins, binding specifically to integrin α1β1 and is classified as small and monomeric (Figure 1) [38,39,41]. Thus, the variation of this tripeptide sequence on the inhibitory loop is one of the main features responsible for the recognition of a distinct integrin (or group of integrins), which results in distinct biological activities (as seen in Figure 2) [29]. 

Many studies have shown that beyond the tripeptide domain of these molecules, there are other regions essential for the interaction with integrin receptors—among them, the C-terminal region. For instance, deletion or replacement of amino acids at the C-terminal region of echistatin, a disintegrin from *Echis carinatus*, can decrease or block the ability to inhibit the adhesion of VNRC3 or K562 cells to immobilized von Willebrand factor or fibronectin, respectively, showing the relevance of the C-terminus for its recognition [42].

## 2. Recombinant Disintegrins

Purification of disintegrins from crude snake venom is a laborious process. Moreover, it yields only a small amount of the protein, sometimes with mixtures of different disintegrins, resulting in limitation of studies on its activity and structure-function relationships. Thus, it is important to produce these proteins in high quantity and good quality in the recombinant form [43]. Furthermore, the use of genetic engineering opens the door to different strategies for studying the structure-activity relationships, e.g., by site-directed mutagenesis, deletion or insertion of amino-acid residues or domains, etc. NMR studies provide high-resolution structure of disintegrins expressed with high yield [44,45]. In addition, studies with recombinant disintegrins have been fused to Enhanced Green Fluorescent Protein (EGFP) for use as molecular biomarkers [46,47], or radiolabeled for clinical diagnosis of potential thrombi [48]. Here we present examples of how these technologies have been used to advance our knowledge about these fascinating molecules. 

### 2.1. Disintegrins from Heterologous Expression 

The use of recombinant DNA technology is widespread in many areas, and many disintegrins have been obtained by this method. Among the critical steps required for successful heterologous expression of a protein, the first is to choose the host system. Factors that influence this option include protein characteristics and the resources available in the laboratory [49]. The second step involves the choice of plasmid, which affects the level of expression and solubility of the recombinant protein. Currently, there are plasmids available that allow for cloning in different cloning frames, with and without selective markers and also the presence or absence of fusion tails that increase solubility, improve purification, and facilitate identification or modification of the final product [43,50].

*Escherichia coli* is the most frequently used host for many reasons: ease of growth, rapid accumulation of biomass, adaptability to large-scale use and low cost. Furthermore, the *Escherichia coli* (*E. coli*) system is widely studied and well characterized, and allows easier genetic manipulation and the development of tools that facilitate gene cloning and expression [51]. Historically, some recombinant snake venom toxins were first expressed by this system, such as phospholipase A2 [52], cysteine-rich venom protein [53] and alpha-neurotoxin [54]. These proteins exhibited the same biological activity as native forms. For that reason, most of the recombinant disintegrins have been obtained using *E. coli.* For example RGD disintegrins like rhodostomin from the venom of *Calloselasma rhodostoma* [55], elegantin from the venom of *Trimeresurus elegans* [56], DisBa-01 from the venom of *Bothrops alternatus* [57], r-mojastin 1 from the venom of *Crotalus s. scutulatus* [58], salmosin from the venom of *Agkistrodon halys brevicaudus* [59] and others (Table 1). Other non-RGD disintegrins were obtained by this host system: obtustatin (KTS) from the venom of *Vipera lebetina obtusa* [60], jerdostatin (RTS) from the venom of *Protobothrops jerdonii* [61], acocostatin (ECD) from the venom of *Agkistrodon c. contortrix* [62], rubistatin (MVD) from the venom of *Crotalus r. ruber* [63].

Different approaches related to the mode of gene expression in *E. coli* can be used to express the desired protein, depending on the information presented in the vector [64]. The production of recombinant proteins expressed in the cytoplasm of *E. coli* is widely used and allows one to obtain proteins with high yield [65]. However, there are some drawbacks to the intracellular production of heterologous proteins in bacteria. Expressing proteins in the cytoplasm of *E. coli* may limit the correct formation of disulfide bonds [66]. The enzymes thioredoxin reductase and glutaredoxin reductase present in the cytoplasm reduce the disulfide bridges of the polypeptide in formation [67,68]. To proteins such as disintegrins, which are rich in cysteine residues, this may lead to low biological activity, protein instability and insolubility [45]. Furthermore, the oxidizing environment in cytoplasm is unavailable, which may result in the production of misfolded recombinant proteins that form insoluble cytoplasmic aggregates known as “inclusion bodies” [69,70]. Although only the recombinant disintegrins DisBa-01 [71] and salmosin [72] were described to form inclusion bodies. 

These insoluble aggregates consist primarily of recombinant proteins devoid of biological activity [73]. To recover these proteins with native conformation, it is necessary to solubilize and refold the proteins from inclusion bodies. Redox reactions can be useful during renaturation when the protein contains disulfide bonds [70]. However, the recovery process requires the use of denaturing agents and laborious steps of refolding. The disadvantage of this process is that it may lead to intermediate aggregates, thus limiting the yield of recombinant protein due to poor recovery [70]. On the other hand, protein expression in the form of inclusion bodies can offer the advantage of an easy isolation of homogeneous recombinant proteins with high levels of expression. Moreover, inclusion bodies confer resistance to proteolytic degradation [73]. Alternative steps to improve recover of proteins in their bioactive form from inclusion bodies can be used. These alternatives include the use of mild solvents instead of denaturing agents in the solubilization step [74,75] and slow dilution as an alternative to dialysis in the refolding step [76].

Different strategies for expression can sometimes overcome these limitations related to disulfide-bond formation or problems with inclusion bodies. Proteins can be expressed by secretion to the periplasmic space of bacteria, where there are cell envelope proteins that favor the formation of disulfide bonds [109,110]. This is a suitable strategy for expression of cysteine-rich proteins. Furthermore, this strategy increases protection against degradation by proteases in the cytoplasm, which reflect on the stability and solubility of protein, and facilitates purification [76]. Despite these advantages, there is also limitations to this mode of expression. The yields obtained by this approach tend to be less than those obtained by expression in the cytoplasm [65,111]. Additionally, there are reports of problems regarding protein stability and solubility when using this strategy [65,112,113]. Expression of proteins to the periplasm is less usual than expression to the cytoplasm, possibly due to the low yield. This problem can be caused by the physical limitation of this environment, which is located between the cell wall and the outer membrane [64]. In fact, disintegrins are usually expressed in the cytoplasm of the bacteria. 

While recombinant proteins can be secreted into the periplasmic space in *E. coli*, it is rare to encounter *E. coli*-secreted proteins in the extracellular medium. This can be achieved using the proper fusion tags for expression to the growth medium [114,115,116,117]. The advantages of this mode of expression are similar to those in periplasmic production and can be an alternative to avoid problems with inclusion bodies [76,118].

Another strategy is to use *E. coli* strains that create oxidative conditions in the cytoplasm (e.g., Shuffle and Origami), due to the presence of mutated thioredoxin reductase (*trxB*) and glutaredoxin reductase (*gor*) genes [108,119]. This type of strain was used for the expression of vicrostatin, resulting in the successful expression of this protein (Table 1).

The choice of plasmid depends on the protein characteristics. One of the common choices is a vector that contains a six-histidine-tag, such as pET28a, pET 39b, and pET32a (+) vectors. Histidine tags are useful because of their small size and the diversity of available purification systems, using a metallic ion as resin [71,87,91]. Note, however, that plasmids that allow expression of the recombinant protein only when it is fused to a histidine tag, such as the pET28a vector, in general do not contribute to a better solubility of the expressed protein [120]. In fact, some studies have reported recombinant proteins expressed in the insoluble fraction such as DisBa-01, being recovered by use of denaturing conditions [71,120]. The protein must be extracted from the insoluble fraction with its biological activity intact, and if it is necessary to use methods of protein refolding for this purpose, they can be time-consuming and may be unsuccessful [120].

Even though the *E. coli* system offers many advantages, some proteins can be expressed with an incorrected fold that may result in their insolubility. To solve these problems, a strategy to increase the solubility is to use plasmids that contain tag tails, such as glutathione S-transferase (GST-tag), thioredoxin or SUMO (Small Ubiquitin-like Modifier) [121].

Numerous disintegrins have been cloned and expressed using GST expression vectors, such as the pGEX vectors series, a system that results in a recombinant fusion protein with a GST-tag that helps in the correct folding and solubility of the protein. Around 23 disintegrins were fused with GST-tag (Table 1), including rhodostomin [55,94,95,96], echistatin [58], bitistatin [79], bothrostatin [80], mojastin 1 and viridistatin 2 [36,58,101,102,103].

For disintegrin expression, other plasmids have been adopted to improve protein solubility. Among them are pET 39b (+), which expresses a DsbA, is a bacterial thiol-disulfide oxidoreductase, and pET32a (+), which expresses a thioredoxin. Both promote disulfide bonds, contributing to the solubility of the expressed protein, such as eristostatin [87] and jerdostatin [61].

Many plasmids have an incorporated tag. Since this may interfere with the biological activity of the protein, the cleavage of these tags is required after expression and purification. There are many sites of cleavage that may be integrated to provide a correct cleavage of the fusion tag site and to avoid leaving extraneous amino acids in the expressed protein. For example, there are sequences that are specific for enterokinase or thrombin. Incorporating SUMO as a fusion protein allows the use of SUMO protease, which does not require extra amino acids to be expressed since it recognizes the tertiary structure of SUMO protein [61,80,93,122].

Unlike *E. coli*, *Pichia pastoris* is a simple eukaryote, capable of performing post-translational modifications, such as disulfide-bridge formation, glycosylation and proteolytic processing [64]. Recombinant proteins expressed in *P. pastoris* may remain in the cytoplasm or be secreted. Proteins expressed in the cytoplasm are exposed to several proteases that degrade exogenous proteins. This problem can be avoided by the use of some *P. pastoris* strains with protease genes mutated, such as the gene *PEP4* and *PRB1*, thus decreasing the levels of proteolysis [123,124]

The formation of disulfide bonds generally occurs in the proteins that will be secreted. This requires that the mRNA contains a signal sequence, which directs the proteins to the endoplasmic reticulum, where they are folded correctly. After proper folding, the proteins are directed to the Golgi complex, where the signal peptide is cleaved; then they are packaged into vesicles to be secreted [125]. Proteins expressed to the extracellular environment can be exposed to proteinase activity. This occurs due to cell lysis, which is caused by high cell density in the medium, promoting protease release [123,124].

Yeast has been useful as a host system to obtain some of the disintegrins. The expression system with yeast (*Pichia pastoris*) has been used for industrial production of proteins since the 1990s, due to its advantages over expression in eukaryotes. Moreover, it is easily manipulated genetically, enables the targeting of expressed protein for secretion, and has a high yield, reaching 20 g/L of culture [123]. In this expression system, there are two kinds of vectors, for intracellular or secreted expression. The latter has been the most used for disintegrin expression. The vector *pPIC9* was used to express the RGD disintegrins saxatilin, from the venom of *Gloydius saxatilis* [123,126] and salmosin from the venom of *Agkistrodon halys brevicaudus* [127]. Leucurogin (ECD), a non-RGD disintegrin from the venom of *Bothrops leucurus*, was also expressed in this vector (Table 2) [128].

### 2.2. Mutagenesis of Disintegrins

Site-directed mutagenesis is a technique used for the study of genes, protein structure and function by modification of the vector sequence to obtain a mutated recombinant protein. This method provides essential information to understand the role of a specific amino-acid residue or protein sequence [137]. In the field of disintegrins, this approach has been widely used to improve the interaction with integrin receptors or to elucidate the importance of amino acids adjacent to or within the motif of interaction, the hairpin loop.

The role of the RGD motif of RGD-containing proteins has been studied and the arginine and aspartic acid residues were found to be critical for the biological activity [131]. The replacement of these two amino acids decreases the relative specificity for binding to integrin αIIbβ3 and the ability to inhibit platelet aggregation [131,138], or results in loss of ability to inhibit platelet aggregation or cell attachment assays [94]. Within the tripeptide, aspartic acid proved to be the most critical residue in its capacity to bind to integrins, as shown by site-directed mutagenesis of kistrin and rhodostomin [94,131,138]. Other mutagenesis studies of non-RGD-containing disintegrins have shown that the ability of jerdostatin, an RTS-containing protein, to inhibit integrin α1β1 is impaired due to replacement of arginine by lysine within the loop [61]. More recently, the RTS-loop of wild-type jerdostatin was used to construct different hybrids with ocellatusin as template in order to confer an α1β1 binding specificity on the mutants. However, the mutants were not capable of block the binding of soluble α1β1 to collagen type IV fragment CB3, suggesting that other factors besides the hairpin loop are required to confer affinity for α1β1 in disintegrins of the RTS group [91].

Despite the importance of disintegrin motifs, specific amino-acid residues flanking the tripeptide sequence also have an important role related to the specificity and capability of interaction with integrins. Several mutated proteins have been used as models to elucidate the importance of these adjacent residues. An example from rhodostomin was an appraisal of the influence of the proline residue adjacent to the RGD motif at the N-terminal side of this disintegrin based on replacing the proline with alanine. The mutated disintegrin had a greater affinity for integrin α5β1 compared to the wild-type rhodostomin, attributed to the increased flexibility of the RGD loop, as shown by NMR studies [139].

Rahman et al. [56], studied the importance and function of amino acids flanking the RGD sequence of recombinant elegantin, from the venom of *Trimeresurus elegans*. Elegantin has a greater inhibitory effect on platelet adhesion to fibronectin, while kistrin has a preference for inhibiting platelet adhesion to fibrinogen [56]. Changes in the amino-acid residues adjacent to RGD in elegantin (^50^ARGDNP), to residues based on the sequence of kistrin (^49^PRGDMP), have shown that a single amino-acid substitution of N54M was capable of impairing platelet adhesion to fibrinogen, making elegantin similar to kistrin. This modification allows the mutated elegantin to be more potent against αIIbβ3 than the wild-type elegantin, since the mutant elegantin was able to inhibit platelet adhesion not only in fibronectin but in both immobilized substrates. Another mutant, with simultaneous mutations A50P and N54M, retained its inhibitory capability against fibrinogen; however, the ability to inhibit platelet adhesion to fibronectin was decreased by the proline substitution, as for kistrin [56]. 

Other recombinant disintegrins showed different biological activities, due to mutations in the C-terminal domain of these peptides [36]. For example, r-mojastin (^51^RGDWN), as cited, is supposed to show a high affinity for integrin αIIbβ3, due to the tryptophan adjacent to the motif [36]. To alter or diminish the biological activity of r-mojastin, Seoane et al. produced a truncated r-mojastin and two mutant peptides with one (W54N) or two (W54D/N55M) mutations in order to compare their platelet aggregation activity and induction of apoptosis in tumor cells [101]. Neither the truncated r-mojastin nor the mutant W54N lost the platelet aggregation inhibitory activity. However, the mutant with two amino-acid substitutions lost the platelet aggregation inhibitory activity [101] and was the only one able to induce apoptosis of tumor cells. Although the mojastin mutant with an additional aspartic acid was able to induce apoptosis of tumor cells, the mere amino-acid substitution by aspartic acid was not enough, per se, to maintain the pro-apoptotic activity. Recently, Ramos et al. showed that among six mojastin mutants with a second aspartic acid, half of them were able to induce apoptosis in melanoma cells [104]. 

The same group has studied the influence of specific mutations on the carboxyl side of the RGD motif of r-mojastin to produce a potent anticancer peptide. The results of replacing the first or second residues following the carboxy-terminus of the RGD with methionine conferred to some of the main mutants potent inhibitory activity on platelet aggregation, angiogenesis and cell migration [140]. These results are consistent with other studies in which this amino-acid substitution induced different biological activities, including potent platelet-aggregation inhibition [56] and increased inhibitory effect on the adhesion of αvβ3-transfected cells to vWF [42]

Hong et al. studied the role of each disulfide bond on structure and function of the saxatilin disintegrin, replacing the cysteines by serines [126]. They evaluated four disintegrins: SaxAB (mutation in Cys6–Cys15 and Cys8–Cys16), SaxC (mutation in Cys21–Cys34), SaxD (mutation in Cys29–Cys59) and SaxF (mutation in Cys47–Cys66), in comparison with saxatilin and saxatilin pretreated with dithiothreitol (DTT). The experimental tests demonstrated that the disulfide bonds Cys47–Cys66 and Cys29–Cys59 are critical for maintaining the activity of the molecule. SaxD and SaxF had similar activity to the disintegrin incubated with DTT, showing the importance of the correct formation of the RGD loop for the activity of the disintegrin. Moreover, SaxAB showed similar activity to wild-type disintegrin, demonstrating that presence of the Cys6-Cys15 bond at the N-terminal is not essential for the activity of this disintegrin. The study also showed that although the Cys21–Cys34 bond does not participate in the formation of the loop, the loss of this disulfide bond decreases the activity of the molecule [126]. 

These studies show that single or multiple substitutions in the residues flanking the hairpin loop sequence have an important impact on the specificity of integrin recognition.

### 2.3. Chimeric Disintegrins

Another useful approach to evaluating structure-function relationships and properties of disintegrins is to produce chimeric peptides. These compounds are constructed from two or more different proteins or sequences, which results in a unique peptide combination with modified protein stability, affinity or function [141].

These hybrid molecules have been studied since 1999 by Wierzbicka-Patynowski et al., contributing to our understanding of the importance of the hairpin loop and the C-terminal region of echistatin, as well as the amino acids that contribute to the ability to recognize integrin receptors [42]. This group investigated the effect of hybrid molecules of echistatin for their inhibitory effect on adhesion of VNRC3 cells to immobilize vWF via αvβ3 blockage. They also examined the inhibition by echistatin hybrids on adhesion of K562 cells to fibronectin via α5β1 blockage. The hybrid mutant of echistatin R22V/D27W/M28N, in which the amino-acid residues of the C-terminal portion (HKGPAT) were replaced by three residues from the C-terminus of eristostatin (WNG), resembled eristostatin with respect to its interaction with the αvβ3 receptor. Furthermore, the effect of the hybrid mutants of echistatin, M28L (1–43)-WNG and R22V/D27W/M28N (1–43)-WNG, resemble eristostatin in their interaction with the α5β1 receptor.

The construction of vicrostatin used this approach. This chimeric peptide carries the HKGPAT amino-acid sequence from echistatin, and these six amino acids replace the C-terminal tail of contortrostatin (*Agkistrodon contortrix contortrix*). This sequence was able to improve the affinity of this mutant for the integrin α5β1 receptor, while still preserving the correct fold. This new chimeric disintegrin showed in vitro anti-migration/anti-invasion properties [107].

The construction of chimeric proteins can also add new functions by adding specific sequences to proteins. Jing and Lu [142], produced a chimera using an eight amino-acid peptide sequence containing the KGD motif, originally from the disintegrin barbourin. This region was selected to replace the loop sequence from the C-peptide of proinsulin. Thus, researchers obtained a new, potent non-immunogenic peptide with specific anti-thrombotic action. This KGD-proinsulin was capable of inhibiting platelet aggregation and recognizing αIIbβ3 receptors while having no hormone activity and no immunogenicity against the human body [142].

## 3. Medical Relevance of Snake Venom Disintegrins

### 3.1. Disintegrins as Modulators of Integrin Activity

Most snake venom disintegrins containing the RGD or related motif potently block integrin αIIbβ3. The potential clinical use of disintegrins as anti-thrombotic agents appeared with the study of platelets in the context of these peptides. Trigramin, isolated from *Trimeresurus gramineus* venom, was first described as a potent aggregation inhibitor for ADP-induced aggregation of human platelets. Thus, the fact that trigramin interacts specifically with integrin αIIbβ3 should make it a useful tool for blocking platelet aggregation activity [17]. Mojastin 1 and contortrostatin, two disintegrins containing the RGD motif, were able to inhibit platelet aggregation with a high affinity toward integrin αIIbβ3 [143,144]. 

With advances in the study of disintegrins, synthetic integrin antagonists have been developed, including Eptifibatide, a cyclic heptapeptide, and Tirofiban, a small non-peptide molecule. These drugs were developed based on the disintegrin structures of barbourin and the RGD disintegrin echistatin, respectively [145,146]. Curiously, some disintegrins have been found to activate rather than inhibit platelets. Rhodostomin, an RGD disintegrin identified in the crude venom of *Calloselasma rhodostoma*, was capable of inducing platelet activation and platelet shape change [94,95]. 

Most disintegrins that recognize and block integrin αIIbβ3 can bind to αvβ3 as well. Contortrostatin was found to recognize αvβ3 and was widely characterized in vitro and in vivo using different tumor cells, showing a promising ability to inhibit adhesion, migration, angiogenesis and tumor progression [147,148,149]. This RGD disintegrin affected angiogenesis by inhibiting the adhesion of endothelial cells to vitronectin, as well as by inhibiting migration and invasion of these cells in vitro with high affinity toward αvβ3 [150]. Other disintegrins containing the KTS (Lys-Thr-Ser) motif were found to impair angiogenesis. Obtustatin inhibited the development of new vessels in chorioallantoic membrane tissue and tumor growth in lung carcinoma, and viperistatin potently inhibited adhesion and migration [151,152]. Based on the structure of these disintegrins, the cyclic peptides vimocin and vipadin were constructed with the tripeptide sequence KTS, and were shown to inhibit proliferation, migration and angiogenesis in vitro and in vivo with high affinity toward integrins α1β1 and α2β1 [153]. This same group showed that vipegetide, a linear peptide containing the sequence WKTSRTSHY of viperistatin, is able to inhibit platelet aggregation. This vipegetide effect arises mainly from its capacity to bind to integrin α2β1, which is expressed on platelet membranes. The authors observed that this peptide can inhibit platelet aggregation induced by adenosine diphosphate and collagen not only in platelet-rich plasma but also in whole human blood, which makes vipegetide a potent anti-aggregation molecule [154].

Interestingly, the role of disintegrins as agonists rather than antagonists of integrin receptors has been further reported. Jarastatin, an RGD disintegrin isolated from *Bothrops jararaca* venom, induces neutrophil chemotaxis, and this effect is dependent on the activation of by integrin receptors α_M_β2 and α5β1 [7,155,156]. Flavoridin, an RGD disintegrin from *Trimeresurus flavoviridis*, binds quite selectively to α5β1 and, despite this, does not interfere with neutrophil functions, nor does it activate integrin signaling or NF-kB signaling in T lymphocytes [157]. Other disintegrins incorporating a different tripeptide domain such as VLO5 (VGD/MLD) and obtustatin have also been shown to interfere with distinct cellular functions. When VLO5 binds to the integrin α9β1 occurs delays of apoptosis in neutrophils [158]. Obtustatin, which selectively binds to α1β1, the collagen receptor, inhibits the production of reactive oxygen species and proliferation of vascular smooth muscle cells [159,160]. 

### 3.2. Recombinant and Chimeric Disintegrins in Preclinical Studies

The ubiquitous presence and the diverse roles of integrins point to an attractive possibility for the use of disintegrins, more specifically the recombinant ones, in therapeutics. Due to their ability to inhibit adhesion, disintegrins may represent potential tools for cancer therapy, since adhesion is a critical step in angiogenesis. 

For example, the recombinant RGD disintegrins r-mojastin and r-viridistatin, due to their ability to bind to integrins αvβ3 and αvβ5, have shown potent anti-angiogenic properties, both in vivo and in vitro. These disintegrins inhibited not only the adhesion of endothelial cells to fibronectin but also their migration, proliferation, and tube formation [37]. Corroborating this idea, the same group had previously shown that r-mojastin was able to inhibit tumor cell adhesion, migration, and invasion [102].

DisBa-01 is another well-studied recombinant RGD disintegrin that has also been pointed out for possible application in the treatment of different diseases, such as cancer and incision hernia (IH). Ramos et al. showed that in vivo blockage of αvβ3 by DisBa-01 inhibited bFGF-induced angiogenesis in a Matrigel plug and also inhibited lung metastasis of melanoma cells in vivo [71]. Evidence has shown that DisBa-01 could be of relevance for the treatment of fibroproliferative diseases since it inhibited both angiogenic and inflammatory/fibrogenic components in fibrovascular tissue [81]. Recently, Oliveira et al. demonstrated that DisBa-01 facilitated wound healing in an IH mouse model, suggesting DisBa-01 as a new therapeutic strategy for IH treatment [82].

Interestingly, studies linking a crosstalk between integrins and TLR receptors during sepsis were performed using rhodostomin, a selective αvβ3-binding recombinant disintegrin. Hsu et al. showed that treatment with this disintegrin attenuated LPS-induced endotoxemia in vivo, an effect attributed to its anti-inflammatory effects on monocytes/macrophages, via αvβ3 blockage and attenuation of TLR4 activation [161]. More recently, a model of sepsis induced by caecal ligation and puncture was used to show that recombinant rhodostomin reduced the release of proinflammatory cytokines and chemokines, increasing the survival rate of the animals [132]. The same authors also showed that, besides TLR4 inhibition, recombinant rhodostomin also impaired the crosstalk between integrin αvβ3 and TLR2. The authors suggested αvβ3 as one of the key targets in sepsis and inferred that recombinant rhodostomin could be useful in treating sepsis.

Leucurogin, the recombinant disintegrin-like ECD described by Higuchi et al. [128], was shown to inhibit Ehrlich tumor growth by more than 50%. The authors believe that this inhibitory effect could be at least partially explained by the effects of leucurogin on vascularization in vivo, acting as a potent angiogenesis inhibitor. Recombinant salmosin is also able to inhibit B16BL6 mouse melanoma cell migration and neovascularization in vivo [162].

One striking example of the utility of hybrids is the already cited chimeric recombinant disintegrin vicrostatin, which was generated to potentiate the anticancer activity of echistatin and contortrostatin peptides that formed the chimera. Vicrostatin significantly inhibits MDA-MB-231 or MDA-MB-435 breast cancer cell migration in vitro. The same study showed that vicrostatin inhibits tubulogenesis and migration of human umbilical vein endothelial cells (HUVEC) [108].

This same group has been working on the development of a clinically relevant delivery method for disintegrins. In 2004, Swenson et al. demonstrated that a liposomal formulation of native contortrostatin could delay tumor growth by reducing the microvascular density in an animal cancer model [163]. Moreover, using liposomal delivery, they provided evidence that disintegrins can be safely and efficiently administered intravenously and that they passively accumulate at the tumor site [163]. In vivo results using liposome-packaged vicrostatin provided further support for its anti-tumoral effect, showing its ability to induce tumor cell apoptosis, inhibit tumor growth and significantly prolong survival of mice [108]. 

Disintegrins present important properties for therapeutic use in different models, as shown by many studies. Nevertheless, because of their low molecular weight, they are potentially challenging to formulate due to their rapid renal clearance [163]. To enhance retention of anticancer agent disintegrin vicrostatin, Janib et al. performed a fusion of vicrostatin and a high-molecular-weight elastin-like polypeptide, A192 [164]. The fusion protein (A192-VCN) has a reduced renal clearance, and like vicrostatin, A192-VCN retains its specificity, binding to MD-MBA-435, MD-MBA-231, and HUVEC in vitro [164]. These findings open a new perspective for the use of disintegrins as therapeutic molecules in cancer.

## 4. Concluding Remarks

Recombinant disintegrins are essential tools for understanding the structure-activity relationships of integrins and their ligands; furthermore, they provide new insights and new structural variants that potentially can overcome limitations such as those associated with delivery and clearance. Nevertheless, as we can see, the very diversity of biological models used in the study of recombinant disintegrins can lead to misinterpretation of data and specificity. It is essential to establish key models to be used as standard assays. The ultimate aim would be to determine the specificity of disintegrins and their derivatives. In the future, this knowledge will allow the development of new drugs while avoiding further side effects.

The relevance of disintegrins, and especially chimeric ones, has been greatly expanded in recent studies. Their targets now include not only traditional targets such as thrombosis (as demonstrated by the development of Integrillin) but also cancer, wound healing, inflammation and other pathologies where integrins play a central role. Recombinant engineering provides new tools for construction of molecules leading to powerful drugs with the ideal characteristics of specificity, delivery, and clearance. 

## Figures and Tables

**Table 1:** Recombinant disintegrins expressed using different plasmids and *E. coli* strain cells.

Disintegrin	Motif	Snake Venom **	Plasmid	Origin of Gene	Expressed	Assay with Fusion Protein or Tag	Yield (mg/L)	Integrin	Ref.
Acocostatin	ECD	*A. c. contortrix*	pGEX-KG	cDNA	*E. coli BL21*	GST	NM	NE	[62]
Adinbitor	RGD	*A. h. b. stejneger*	pET23b	cDNA	*E. coli BL21*	His-Tag	NM	NE	[51,77,78]
Bitistatin	RGD	*B. arietans*	pGEX-KT, pET-5a	S	*E. coli BL21*	GST, no	1–2 (free) 12 ± 3 (free)	αIIbβ3 *	[79]
Bothrostatin	RGD	*B. jararaca*	pGEX-4T	cDNA	*E. coli BL21*	GST, no	6 (free)	NE	[80]
DisBa-01	RGD	*B. alternatus*	pET28a	cDNA	*E. coli BL21*	His-Tag	NM	αIIbβ3, αvβ3	[57,71,81,82,83,84]
Echistatin and mutants	RGD	*E. carinatus*	pGEX-KG	S	*E. coli BL21*	No	NM	αIIbβ3, αvβ3 *, α5β1 *	[42,85,86]
Elegantin and mutant	RGD	*T. elegans*	pGEX-3X	S	*E. coli (DE3) pLysS*	GST	NM	αIIbβ3, α5β1	[56]
Eristostatin and mutants	RGD	*E. macmahoni*	pGEX-KG, pET 39b	S	*E. coli BL21*	No	NM	αIIbβ3 *, αvβ3 *, α5β1 *	[42,87,88,89]
Insularin	RGD	*B. insularis*	pGEX-4-T1	cDNA	*E. coli BL21*	GST	50 (fused)	αIIbβ3 *, αvβ3 *	[90]
Jerdostatin and mutant	RTS	*P. jerdonii*	pET32a	cDNA	*E. coli Origami B*, *E. coli BL21*	No	0.5–2	α1β1	[61,91,92]
Neuwiedin	RGD	*B. neuwiedi*	pMST3	cDNA	*E. coli C43*	No	8	αIIbβ3 *, αvβ3 *	[93]
Obtustatin and mutant	KTS	*V. l. obtusa*	pGEX-4-T1	S	*E. coli BL21*	No	NM	α1β1	[60]
Rhodostomin and mutant	RGD	*C. rhodostoma*	pGEX-2KS, pGEX-2T	S cDNA	*E. coli K38*, *E. coli RR1*, *E. coli DH5α*	GST	NM	αIIbβ3, αvβ3, α4β1 α5β1	[55,94,95,96,97]
Rubistatin	MVD	*C. r. ruber*	pET32b	cDNA	*E. coli origami2*	No	NM	NE	[63]
r-Cam-dis	RGD	*C. adamanteus*	pET-43.1a, pGEX-4T-1	cDNA	*E. coli BL21*	No	1	αIIbβ3, αvβ3, αvβ5, αvβ6, α2β1, α6β1	[98,99]
r-colombis-tatin	ECD	*B. colombiensis*	pGEX-4-T1	cDNA	*E. coli BL21*	No	NM	NE	[100]
r-mojastin 1 and mutant	RGD	*C. s. scutulatus*	pGEX-4-T1, pGEX-KG	cDNA	*E. coli BL21*	GST, no	0.8 (free)	αIIbβ3 *, αv *, αvβ3 *, αvβ5 *, α3β1 *, α6 *, β1 *, β3 *	[37,58,101,102,103,104]
r-ocellatusin and mutant	RGD RTS	*E. ocellatus*	pET32a	cDNA	*E. coli BL21*	No	0.5–1	NE	[91]
r-viridistatin 2	RGD	*C. v. viridis*	pGEX-4-T1	cDNA	*E. coli BL21*	No	NM	αvβ3, αvβ5, α3β1	[37,103,105]
Salmosin 1	RGD	*A. h. brevicandus*	pMA-PRK153, ΔpMA	cDNA	*E. coli MC1061*	PRK	NM	αIIbβ3, αvβ3	[59,72,106]
Vicrostatin	RGD	*chimeric recombinant*	pET32a	NM	*Origami B (DE3) pLysS*	No	20	αvβ3, αvβ5, α5β1	[107,108]

Abbreviations: S: Synthetic; NM: Not mentioned; NE: Not evaluated; (*) means specificity not confirmed; (**) Origin of wild-type disintegrin.

**Table 2:** Recombinant disintegrins expressed using different plasmids and *Pichia pastoris* strain cells.

Disintegrin	Motif	Snake Venom **	Plasmid	Origin of Gene	Expressed	Assay with Fusion Protein or Tag	Yield (mg/L)	Integrin	References
Albolatin	KGD	*T. albolabris*	*pPICZα A*	cDNA	*P. pastoris X33*	His-Tag	3.3	NE	[129]
Echistatin (Ech)	RGD	*Echis carinatus*	*pPICZα A*	S	*P. pastoris X33*	His-Tag	2–7	αIIbβ3	[44]
Leucurogin	ECD	*Bothrops leucurus*	*pPIC9*	cDNA	*P. pastoris X33*	No	NM	NE	[127]
Rhodostomin mutant	RGD	*Calloselasma rhodostoma*	*pPICZαA*	NM	*P. pastoris X33*	No	NM	NE	[130]
Rhodostomin and mutant	RGD	*Calloselasma rhodostoma*	*pPICZαA*	S NM	*P. pastoris X33*	No	10–25	αIIbβ3, α5β1 αvβ3	[44,131,132,133]
Salmosin 1	RGD	*Agkistrodon h. brevicandus*	*pPIC9*	cDNA	*P. pastoris*	No	NM	αvβ3	[127]
Saxatilin and mutant	RGD	*Gloydius saxatilis*	*pPIC9*	cDNA	*P. pastoris GS115*	No	150	αIIbβ3, αvβ3	[126,134,135,136]

Abbreviations: S: Synthetic; NM: Not mentioned; NE: Not evaluated; (**) Origin of wild-type disintegrin.

## References

[1] Tcheng J.E., Harrington R.A., Kottke-Marchant K., Kleiman N.S., Ellis S.G., Kereiakes D.J., Mick M.J., Navetta F.I., Smith J.E., Worley S.J. (1995). Multicenter, Randomized, Double-Blind, Placebo-Controlled Trial of the Platelet Integrin Glycoprotein IIb/IIIa Blocker Integrelin in Elective Coronary Intervention. Circulation.

[2] Gould R.J., Polokoff M.A., Friedman P.A., Huang T.F., Holt J.C., Cook J.J., Niewiarowski S. (1990). Disintegrins: A family of integrin inhibitory proteins from viper venoms. Proc. Soc. Exp. Biol. Med..

[3] McLane M., Sanchez E., Wong A., Paquette-Straub C., Perez J. (2004). Disintegrins. Curr. Drug Target Cardiovasc. Hematol. Disord..

[4] Phillips D.R., Jennings L.K., Edwards H.H. (1980). Identification of membrane proteins mediating the interaction of human platelets. J. Cell Biol..

[5] Boudreau N.J., Jones P.L. (1999). Extracellular matrix and integrin signalling: The shape of things to come. Biochem. J..

[6] Hynes R. (1987). Integrins: A family of cell surface receptors. Cell.

[7] Coelho A.L., de Freitas M.S., Oliveira-Carvalho A.L., Moura-Neto V., Zingali R.B., Barja-Fidalgo C. (1999). Effects of jarastatin, a novel snake venom disintegrin, on neutrophil migration and actin cytoskeleton dynamics. Exp. Cell Res..

[8] Marcinkiewicz B.C., Vijay-Kumar S., Mclane M.A., Niewiarowski S. (1997). Significance of RGD Loop and C-Terminal Domain of Echistatin for Recognition of αIIbβ3 and avβ3 Integrins and Expression of Ligand-Induced Binding Site. Blood.

[9] Calvete J.J. (2013). The continuing saga of snake venom disintegrins. Toxicon.

[10] Marcinkiewicz C. (2013). Applications of snake venom components to modulate integrin activities in cell-matrix interactions. Int. J. Biochem. Cell Biol..

[11] Macêdo J.K.A., Fox J.W., Castro M.D.S., Kele J., Macêdo A., Fox J.W., Castro M.D.S. (2015). Disintegrins from snake venoms and their applications in cancer research and therapy. Curr. Protein Pept. Sci..

[12] Fox J.W., Serrano S.M.T. (2009). Timeline of key events in snake venom metalloproteinase research. J. Proteom..

[13] McLane M.A., Marcinkiewicz C., Vijay-Kumar S., Wierzbicka-Patynowski I., Niewiarowski S. (1998). Viper Venom Disintegrins and Related Molecules. Exp. Biol. Med..

[14] Calvete J.J., Marcinkiewicz C., Monleón D., Esteve V., Celda B., Juárez P., Sanz L. (2005). Snake venom disintegrins: Evolution of structure and function. Toxicon.

[15] Mclane M.A., Joerger T., Mahmoud A. (2008). Disintegrins in health and disease. Front. Biosci..

[16] Huang T.-F., Hsu C.-C., Kuo Y.-J., Teng C., Huang T., Clemetson K., Hynes R., Ginsberg M., Loftus J., Plow E. (2016). Anti-thrombotic agents derived from snake venom proteins. Thromb. J..

[17] Huang T.F., Holt J.C., Lukasiewicz H., Niewiarowski S. (1987). Trigramin: A low molecular weight peptide inhibiting fibrinogen interaction with platelet receptors expressed on glycoprotein IIb-IIIa complex. J. Biol. Chem..

[18] Barczyk M., Carracedo S., Gullberg D. (2010). Integrins. Cell Tissue Res..

[19] Goodman S.L., Picard M. (2012). Integrins as therapeutic targets. Trends Pharmacol. Sci..

[20] Savage B., Saldívar E., Ruggeri Z.M. (1996). Initiation of platelet adhesion by arrest onto fibrinogen or translocation on von Willebrand factor. Cell.

[21] Calvete J.J. (1999). Platelet integrin GPIIb/IIIa: Structure-function correlations. An update and lessons from other integrins [Review]. Proc. Soc. Exp. Biol. Med..

[22] Nieberler M., Reuning U., Reichart F., Notni J., Wester H.-J., Schwaiger M., Weinmüller M., Räder A., Steiger K., Kessler H. (2017). Exploring the Role of RGD-Recognizing Integrins in Cancer. Cancers.

[23] Horton M.A. (1997). The αvβ3 integrin “vitronectin receptor”. Int. J. Biochem. Cell Biol..

[24] Hynes R.O. (1992). Integrins: Versatility, modulation, and signaling in cell adhesion. Cell.

[25] Lian J., Guoping C., Shapiro S.S., Tran L.-P., Beacham D.A. (1999). Glycoprotein Ibα Can Mediate Endothelial Cell Migration on von Willebrand Factor-Containing Substrata. Exp. Cell Res..

[26] Wong N.C., Mueller B.M., Barbas C.F., Ruminski P., Quaranta V., Lin E.C., Smith J.W. (1998). Alphav integrins mediate adhesion and migration of breast carcinoma cell lines. Clin. Exp. Metast..

[27] Kourtzelis I., Mitroulis I., von Renesse J., Hajishengallis G., Chavakis T. (2017). From leukocyte recruitment to resolution of inflammation: The cardinal role of integrins. J. Leukoc. Biol..

[28] Kini R.M., Evans H.J. (1992). Structural domains in venom proteins: Evidence that metalloproteinases and nonenzymatic platelet aggregation inhibitors (disintegrins) from snake venoms are derived by proteolysis from a common precursor. Toxicon.

[29] Calvete J.J., Moreno-Murciano M.P., Theakston R.D.G., Kisiel D.G., Marcinkiewicz C. (2003). Snake venom disintegrins: Novel dimeric disintegrins and structural diversification by disulphide bond engineering. Biochem. J..

[30] Niewiarowski S., McLane M.A., Kloczewiak M., Stewart G.J. (1994). Disintegrins and other naturally occurring antagonists of platelet fibrinogen receptors. Semin. Hematol..

[31] Lu X., Lu D., Scully M.F., Kakkar V. (2006). V Integrins in drug targeting-RGD templates in toxins. Curr. Pharm. Des..

[32] Müller G., Gurrath M., Kessler H. (1994). Pharmacophore refinement of gpIIb/IIIa antagonists based on comparative studies of antiadhesive cyclic and acyclic RGD peptides. J. Comput. Aided Mol. Des..

[33] Dechantsreiter M.A., Planker E., Mathä B., Lohof E., Hölzemann G., Jonczyk A., Goodman S.L., Kessler H. (1999). N-Methylated Cyclic RGD Peptides as Highly Active and Selective α V β 3 Integrin Antagonists. J. Med. Chem..

[34] Mas-Moruno C., Rechenmacher F., Kessler H. (2010). Cilengitide: The First Anti-Angiogenic Small Molecule Drug Candidate. Design, Synthesis and Clinical Evaluation. Anticancer Agents Med. Chem..

[35] Kapp T.G., Rechenmacher F., Neubauer S., Maltsev O.V., Cavalcanti-Adam E.A., Zarka R., Reuning U., Notni J., Wester H.-J., Mas-Moruno C. (2017). A Comprehensive Evaluation of the Activity and Selectivity Profile of Ligands for RGD-binding Integrins. Sci. Rep..

[36] Scarborough R.M., Rose J.W., Naughton M.A., Phillips D.R., Nannizzi L., Arfsten A., Campbell A.M., Charo I.F. (1993). Characterization of the integrin specificities of disintegrins isolated from American pit viper venoms. J. Biol. Chem..

[37] Lucena S.E., Romo K., Suntravat M., Sánchez E.E. (2014). Anti-angiogenic activities of two recombinant disintegrins derived from the Mohave and Prairie rattlesnakes. Toxicon.

[38] Walsh E.M., Marcinkiewicz C. (2011). Non-RGD-containing snake venom disintegrins, functional and structural relations. Toxicon.

[39] Bazan-Socha S., Kisiel D.G., Young B., Theakston R.D.G., Calvete J.J., Sheppard D., Marcinkiewicz C. (2004). Structural requirements of MLD-containing disintegrins for functional interaction with alpha 4 beta 1 and alpha 9 beta1 integrins. Biochemistry.

[40] Marcinkiewicz C., Taooka Y., Yokosaki Y., Calvete J.J., Marcinkiewicz M.M., Lobb R.R., Niewiarowski S., Sheppard D. (2000). Inhibitory Effects of MLDG-containing Heterodimeric Disintegrins Reveal Distinct Structural Requirements for Interaction of the Integrin α9β1 with VCAM-1, Tenascin-C, and Osteopontin. J. Biol. Chem..

[41] Kisiel D.G., Calvete J.J., Katzhendler J., Fertala A., Lazarovici P., Marcinkiewicz C. (2004). Structural determinants of the selectivity of KTS-disintegrins for the α1β1 integrin. FEBS Lett..

[42] Wierzbicka-Patynowski I., Niewiarowski S., Marcinkiewicz C., Calvete J.J., Marcinkiewicz M.M., McLane M.A. (1999). Structural requirements of echistatin for the recognition of α(v)β3 and α5β1 integrins. J. Biol. Chem..

[43] Rosano G.L., Ceccarelli E.A. (2014). Recombinant protein expression in Escherichia coli: Advances and challenges. Front. Microbiol..

[44] Chen Y.-C., Cheng C.-H., Shiu J.-H., Chang Y.-T., Chang Y.-S., Huang C.-H., Lee J.-C., Chuang W.-J. (2012). Expression in Pichia pastoris and characterization of echistatin, an RGD-containing short disintegrin. Toxicon.

[45] Guo R.T., Chou L.J., Chen Y.C., Chen C.Y., Pari K., Jen C.J., Lo S.J., Huang S.L., Lee C.Y., Chang T.W. (2001). Expression in Pichia pastoris and characterization by circular dichroism and NMR of rhodostomin. Proteins Struct. Funct. Genet..

[46] Magalhães G.S., Novo J.B., Clissa P.B., Della Casa M.S., Butera D., Da Silva A.M.M. (2012). Engineered mammalian vector to express EGFP-tagged proteins as biomarkers. Mol. Biotechnol..

[47] Butera D., Fontes Piazza R.M., McLane M.A., Chammas R., Da Silva A.M.M. (2005). Molecular engineering of an EGFP/disintegrin-based integrin marker. Toxicon.

[48] Knight L.C., Romano J.E., Bright L.T., Agelan A., Kantor S., Maurer A.H. (2007). Platelet binding and biodistribution of [99mTc]rBitistatin in animal species and humans. Nucl. Med. Biol..

[49] Demain A.L., Vaishnav P. (2009). Production of recombinant proteins by microbes and higher organisms. Biotechnol. Adv..

[50] Young C.L., Britton Z.T., Robinson A.S. (2012). Recombinant protein expression and purification: A comprehensive review of affinity tags and microbial applications. Biotechnol. J..

[51] Terpe K. (2006). Overview of bacterial expression systems for heterologous protein production: From molecular and biochemical fundamentals to commercial systems. Appl. Microbiol. Biotechnol..

[52] Kelley M.J., Crowl R.M., Dennis E.A. (1992). Renaturation of cobra venom phospholipase A2 expressed from a synthetic gene in Escherichia coli. Biochim. Biophys. Acta Protein Struct. Mol. Enzymol..

[53] Chang T.-Y., Mao S.-H., Guo Y.-W. (1997). Cloning and expression of a cysteine-rich venom protein from Trimeresurus mucrosquamatus (Taiwan Habu). Toxicon.

[54] Chang L., Lin J., Wu P., Chang C., Hong E. (1997). cDNA Sequence Analysis and Expression of κ-Bungarotoxin from Taiwan Banded Krait. Biochem. Biophys. Res. Commun..

[55] Chang H.H., Hu S.T., Huang T.F., Chen S.H., Lee Y.H.W., Lo S.C.J. (1993). Rhodostomin, an RGD-Containing Peptide Expressed from a Synthetic Gene in Escherichia coli, Facilitates the Attachment of Human Hepatoma Cells. Biochem. Biophys. Res. Commun..

[56] Rahman S., Aitken A., Flynn G., Formstone C., Savidge G.F. (1998). Modulation of RGD sequence motifs regulates disintegrin recognition of αIIbβ3 and α5β1 integrin complexes. Biochem. J..

[57] Kauskot A., Cominetti M.R., Ramos O.H.P., Bechyne I., Renard J.-M., Hoylaerts M.F., Crepin M., Legrand C., Selistre-de-Araujo H.S., Bonnefoy A. (2008). Hemostatic effects of recombinant DisBa-01, a disintegrin from Bothrops alternatus. Front. Biosci..

[58] Sánchez E.E., Lucena S.E., Reyes S., Soto J.G., Cantu E., Lopez-Johnston J.C., Guerrero B., Salazar A.M., Rodríguez-acosta A., Galán J.A. (2010). Cloning, expression, and hemostatic activities of a disintegrin, r-mojastin 1, from the mohave rattlesnake (Crotalus scutulatus scutulatus). Thromb. Res..

[59] Park D., Kang I., Kim H., Chung K., Kim D.S., Yun Y. (1998). Cloning and characterization of novel disintegrins from Agkistrodon halys venom. Mol. Cells.

[60] Brown M.C., Eble J.A., Calvete J.J., Marcinkiewicz C. (2009). Structural requirements of KTS-disintegrins for inhibition of alpha(1)beta(1) integrin. Biochem. J..

[61] Sanz L., Chen R.-Q., Pérez A., Hilario R., Juárez P., Marcinkiewicz C., Monleón D., Celda B., Xiong Y.-L., Pérez-Payá E. (2005). cDNA Cloning and Functional Expression of Jerdostatin, a Novel RTS-disintegrin from Trimeresurus jerdonii and a Specific Antagonist of the α 1 β 1 Integrin. J. Biol. Chem..

[62] Teklemariam T., Seoane A.I., Ramos C.J., Sanchez E.E., Lucena S.E., Perez J.C., Mandal S.A., Soto J.G. (2011). Functional analysis of a recombinant PIII-SVMP, GST-acocostatin; an apoptotic inducer of HUVEC and HeLa, but not SK-Mel-28 cells. Toxicon.

[63] Carey C.M., Bueno R., Gutierrez D.A., Petro C., Lucena S.E., Sanchez E.E., Soto J.G. (2012). Recombinant rubistatin (r-Rub), an MVD disintegrin, inhibits cell migration and proliferation, and is a strong apoptotic inducer of the human melanoma cell line SK-Mel-28. Toxicon.

[64] Berlec A., Strukelj B. (2013). Current state and recent advances in biopharmaceutical production in Escherichia coli, yeasts and mammalian cells. J. Ind. Microbiol. Biotechnol..

[65] Latifi A.M., Khajeh K., Farnoosh G., Hassanpour K., Khodi S. (2015). The Cytoplasmic and Periplasmic Expression Levels and Folding of Organophosphorus Hydrolase Enzyme in Escherichia coli. Jundishapur J. Microbiol..

[66] Ke N., Berkmen M. (2014). Production of Disulfide-Bonded Proteins in Escherichia coli. Curr. Protoc. Mol. Biol..

[67] Ritz D., Beckwith J. (2001). Roles of Thiol-Redox Pathways in Bacteria. Annu. Rev. Microbiol..

[68] Prinz W.A., Åslund F., Holmgren A., Beckwith J. (1997). The Role of the Thioredoxin and Glutaredoxin Pathways in Reducing Protein Disulfide Bonds in the Escherichia coli Cytoplasm. J. Biol. Chem..

[69] Schein C.H. (1989). Production of Soluble Recombinant Proteins in Bacteria. Nat. Biotechnol..

[70] Fahnert B., Lilie H., Neubauer P. (2004). Inclusion Bodies: Formation and Utilisation. Physiological Stress Response in Bioprocesses. Advances in Biochemical Engineering.

[71] Ramos O.H.P., Kauskot A., Cominetti M.R., Bechyne I., Salla Pontes C.L., Chareyre F., Manent J., Vassy R., Giovannini M., Legrand C. (2008). A novel αvβ3-blocking disintegrin containing the RGD motive, DisBa-01, inhibits bFGF-induced angiogenesis and melanoma metastasis. Clin. Exp. Metast..

[72] Kang I., Lee Y., Kim D. (1999). A Novel Disintegrin Salmosin Inhibits Tumor Angiogenesis A Novel Disintegrin Salmosin Inhibits Tumor Angiogenesis 1. Cancer Res..

[73] Vallejo L.F., Rinas U. (2004). Strategies for the recovery of active proteins through refolding of bacterial inclusion body proteins. Microb. Cell Fact..

[74] Singh S.M., Sharma A., Upadhyay A.K., Singh A., Garg L.C., Panda A.K. (2012). Solubilization of inclusion body proteins using n -propanol and its refolding into bioactive form. Protein Expr. Purif..

[75] Zhuravko A.S., Kononova N.V., Bobruskin A.I. (2015). Features of the solubilization of interferon beta-1B from inclusion bodies. Russ. J. Bioorg. Chem..

[76] Kaur J., Kumar A., Kaur J. (2018). Strategies for optimization of heterologous protein expression in E. coli: Roadblocks and reinforcements. Int. J. Biol. Macromol..

[77] Zhao C., Cui X., Ren F., Zhao B. (2008). rAdinbitor, a novel disintegrin from Agkistrodon halys brevicaudus stejneger inhibits adhesion and proliferation of SMMC-7721 cells. Chin. Ger. J. Clin. Oncol..

[78] Sun M.-Z.Z., Cui Y., Guo C., Zhao B., Liu S. (2015). rAdinbitor, a disintegrin from Agkistrodon halys brevicaudus stejneger, inhibits tumorigenicity of hepatocarcinoma via enhanced anti-angiogenesis and immunocompetence. Biochimie.

[79] Knight L.C., Romano J.E. (2005). Functional expression of bitistatin, a disintegrin with potential use in molecular imaging of thromboembolic disease. Protein Expr. Purif..

[80] Fernandez J.H., Silva C.A., Assakura M.T., Camargo A.C.M., Serrano S.M.T. (2005). Molecular cloning, functional expression, and molecular modeling of bothrostatin, a new highly active disintegrin from Bothrops jararaca venom. Biochem. Biophys. Res. Commun..

[81] Cassini-Vieira P., Deconte S.R.A., Tomiosso T.C., Campos P.P.E., Montenegro C.D.F., Selistre-de-Araújo H.S., Barcelos L.S., Andrade S.P.A., Araújo F.D.A. (2014). DisBa-01 inhibits angiogenesis, inflammation and fibrogenesis of sponge-induced-fibrovascular tissue in mice. Toxicon.

[82] de Oliveira C.R., de Marqueti R.C., Cominetti M.R., Douat E.S.V., Ribeiro J.U., Pontes C.L.S., Borghi-Silva A., Selistre-de-Araujo H.S. (2015). Effects of blocking αvβ3 integrin by a recombinant RGD disintegrin on remodeling of wound healing after induction of incisional hernia in rats. Acta Cir. Bras..

[83] Montenegro C.F., Salla-Pontes C.L., Ribeiro J.U., MacHado A.Z., Ramos R.F., Figueiredo C.C., Morandi V.V.V., Selistre-De-Araujo H.S. (2012). Blocking αvβ3 integrin by a recombinant RGD disintegrin impairs VEGF signaling in endothelial cells. Biochimie.

[84] Ribeiro L.C., Massimino L.C., Durante A.C., Tansini A., Urbaczek A.C., Selistre-de-Araújo H.S., Carlos I.Z. (2014). Recombinant disintegrin targets α(v) β(3) integrin and leads to mediator production. Cell Adhes. Migr..

[85] Hantgan R.R., Stahle M.C., Connor J.H., Lyles D.S., Horita D.A., Rocco M., Nagaswami C., Weisel J.W., McLane M.A. (2004). The Disintegrin Echistatin Stabilizes Integrin αIIbβ3’s Open Conformation and Promotes Its Oligomerization. J. Mol. Biol..

[86] Hantgan R.R., Stahle M.C., Connor J.H., Horita D.A., Rocco M., McLane M.A., Yakovlev S., Medved L. (2006). Integrin αIIbβ3:ligand interactions are linked to binding-site remodeling. Protein Sci..

[87] Tian J., Paquette-Straub C., Sage E.H., Funk S.E., Patel V., Galileo D., McLane M.A. (2007). Inhibition of melanoma cell motility by the snake venom disintegrin eristostatin. Toxicon.

[88] McLane M.A., Zhang X., Tian J., Zelinskas C., Srivastava A., Hensley B., Paquette-Straub C. (2006). Scratching below the surface: Wound healing and alanine mutagenesis provide unique insights into interactions between eristostatin, platelets and melanoma cells. Pathophysiol. Haemost. Thromb..

[89] Hailey S., Adams E., Penn R., Wong A., McLane M.A. (2013). Effect of the disintegrin eristostatin on melanoma-natural killer cell interactions. Toxicon.

[90] Della-Casa M.S., Junqueira-de-Azevedo I., Butera D., Clissa P.B., Lopes D.S., Serrano S.M.T., Pimenta D.C., Magalhães G.S., Ho P.L., Moura-da-Silva A.M. (2011). “Insularin, a disintegrin from Bothrops insularis venom: Inhibition of platelet aggregation and endothelial cell adhesion by the native and recombinant GST-insularin proteins”. Toxicon.

[91] Sanz-Soler R., Lorente C., Company B., Sanz L., Juárez P., Pérez A., Zhang Y., Jin Y., Chen R., Eble J.A. (2012). Recombinant expression of mutants of the Frankenstein disintegrin, RTS-ocellatusin. Evidence for the independent origin of RGD and KTS/RTS disintegrins. Toxicon.

[92] Bolás G., de Rezende F.F., Lorente C., Sanz L., Eble J.A., Calvete J.J. (2014). Inhibitory effects of recombinant RTS-jerdostatin on integrin α1β1 function during adhesion, migration and proliferation of rat aortic smooth muscle cells and angiogenesis. Toxicon.

[93] Lima-dos-Santos I., Della-Casa M.S., Portes-Junior J.A., Calabria P.A.L., Magalhães G.S., Moura-da-Silva A.M. (2015). Characterization of Neuwiedin, a new disintegrin from Bothrops neuwiedi venom gland with distinct cysteine pattern. Toxicon.

[94] Chang H.H., Tsai W.J., Lo S.J. (1997). Glutathione S-transferase-rhodostomin fusion protein inhibits platelet aggregation and induces platelet shape change. Toxicon.

[95] Chang H.H., Lo S.J. (1998). Full-spreading platelets induced by the recombinant rhodostomin are via binding to integrins and correlated with FAK phosphorylation. Toxicon.

[96] Tselepis V.H., Green L.J., Humphries M.J. (1997). An RGD to LDV motif conversion within the disintegrin kistrin generates an integrin antagonist that retains potency but exhibits altered receptor specificity. Evidence for a functional equivalence of acidic integrin- binding motifs. J. Biol. Chem..

[97] Chang H.H., Lin C.H., Lo S.J. (1999). Recombinant rhodostomin substrates induce transformation and active calcium oscillation in human platelets. Exp. Cell Res..

[98] Suntravat M., Jia Y., Lucena S.E., Sánchez E.E., Pérez J.C. (2013). cDNA cloning of a snake venom metalloproteinase from the eastern diamondback rattlesnake (Crotalus adamanteus), and the expression of its disintegrin domain with anti-platelet effects. Toxicon.

[99] Suntravat M., Barret H.S., Jurica C.A., Lucena S.E., Perez J.C. (2015). Recombinant disintegrin (r-Cam-dis) from Crotalus adamanteus inhibits adhesion of human pancreatic cancer cell lines to laminin-1 and vitronectin. J. Venom Res..

[100] Suntravat M., Helmke T.J., Atphaisit C., Cuevas E., Lucena S.E., Uzcátegui N.L., Sánchez E.E., Rodriguez-Acosta A. (2016). Expression, purification, and analysis of three recombinant ECD disintegrins (r-colombistatins) from P-III class snake venom metalloproteinases affecting platelet aggregation and SK-MEL-28 cell adhesion. Toxicon.

[101] Seoane A.I., Tran V.L., Sanchez E.E., White S.A., Choi J.L., Gaytán B., Chavez N., Reyes S.R., Ramos C.J., Tran L.H. (2010). The mojastin mutant Moj-DM induces apoptosis of the human melanoma SK-Mel-28, but not the mutant Moj-NN nor the non-mutated recombinant Moj-WN. Toxicon.

[102] Lucena S., Sanchez E.E., Perez J.C. (2011). Anti-metastatic activity of the recombinant disintegrin, r-mojastin 1, from the Mohave rattlesnake. Toxicon.

[103] Lucena S., Castro R., Lundin C., Hofstetter A., Alaniz A., Suntravat M., Sánchez E.E. (2015). Inhibition of pancreatic tumoral cells by snake venom disintegrins. Toxicon.

[104] Ramos C.J., Gutierrez D.A., Aranda A.S., Koshlaychuk M.A., Carrillo D.A., Medrano R., McBride T.D., U A., Medina S.M., Lombardo M.C. (2016). Functional characterization of six aspartate (D) recombinant mojastin mutants (r-Moj): A second aspartate amino acid carboxyl to the RGD in r-Moj-D_ peptides is not sufficient to induce apoptosis of SK-Mel-28 cells. Toxicon.

[105] Lucena S.E., Jia Y., Soto J.G., Parral J., Cantu E., Brannon J., Lardner K., Ramos C.J., Seoane A.I., Sánchez E.E. (2012). Anti-invasive and anti-adhesive activities of a recombinant disintegrin, r-viridistatin 2, derived from the Prairie rattlesnake (Crotalus viridis viridis). Toxicon.

[106] Kang I.C., Kim D.S., Jang Y., Chung K.H. (2000). Suppressive mechanism of salmosin, a novel disintegrin in B16 melanoma cell metastasis. Biochem. Biophys. Res. Commun..

[107] Minea R., Costa F., Chen C., Markland F.S., Swenson S., Costa F., Chen T.C., Markland F.S. (2005). Development of a novel recombinant disintegrin, contortrostatin, as an effective anti-tumor and anti-angiogenic agent. Pathophysiol. Haemost. Thromb..

[108] Minea R., Helchowski C., Rubino B., Brodmann K., Swenson S., Markland F. (2012). Development of a chimeric recombinant disintegrin as a cost-effective anti-cancer agent with promising translational potential. Toxicon.

[109] Kadokura H., Beckwith J. (2010). Mechanisms of Oxidative Protein Folding in the Bacterial Cell Envelope. Antioxid. Redox Signal..

[110] Yin J., Li G., Ren X., Herrler G. (2007). Select what you need: A comparative evaluation of the advantages and limitations of frequently used expression systems for foreign genes. J. Biotechnol..

[111] Neubauer A., Neubauer P., Myllyharju J. (2005). High-level production of human collagen prolyl 4-hydroxylase in Escherichia coli. Matrix Biol..

[112] Kovalskaya N., Hammond R.W. (2009). Expression and functional characterization of the plant antimicrobial snakin-1 and defensin recombinant proteins. Protein Expr. Purif..

[113] Georgiou G., Telford J.N., Shuler M.L., Wilson D.B. (1986). Localization of inclusion bodies in Escherichia coli overproducing beta-lactamase or alkaline phosphatase. Appl. Environ. Microbiol..

[114] Majander K., Anton L., Antikainen J., Lång H., Brummer M., Korhonen T.K., Westerlund-Wikström B. (2005). Extracellular secretion of polypeptides using a modified Escherichia coli flagellar secretion apparatus. Nat. Biotechnol..

[115] Qian Z.-G., Xia X.-X., Choi J.H., Lee S.Y. (2008). Proteome-based identification of fusion partner for high-level extracellular production of recombinant proteins in Escherichia coli. Biotechnol. Bioeng..

[116] Gao D., Wang S., Li H., Yu H., Qi Q. (2015). Identification of a heterologous cellulase and its N-terminus that can guide recombinant proteins out of Escherichia coli. Microb. Cell Fact..

[117] Schmoldt H.-U., Wentzel A., Becker S., Kolmar H. (2005). A fusion protein system for the recombinant production of short disulfide bond rich cystine knot peptides using barnase as a purification handle. Protein Expr. Purif..

[118] Wan E.W.M., Baneyx F. (1998). TolAIII co-overexpression facilitates the recovery of periplasmic recombinant proteins into the growth medium of Escherichia coli. Protein Expr. Purif..

[119] Lobstein J., Emrich C.A., Jeans C., Faulkner M., Riggs P., Berkmen M. (2012). SHuffle, a novel Escherichia coli protein expression strain capable of correctly folding disulfide bonded proteins in its cytoplasm. Microb. Cell Fact..

[120] Marblestone J.G. (2006). Comparison of SUMO fusion technology with traditional gene fusion systems: Enhanced expression and solubility with SUMO. Protein Sci..

[121] Butt T.R., Edavettal S.C., Hall J.P., Mattern M.R. (2005). SUMO fusion technology for difficult-to-express proteins. Protein Expr. Purif..

[122] Shimokawa-Falcão L., Caporrino M., Barbaro K., Della-Casa M., Magalhães G. (2017). Toxin Fused with SUMO Tag: A New Expression Vector Strategy to Obtain Recombinant Venom Toxins with Easy Tag Removal inside the Bacteria. Toxins.

[123] Ahmad M., Hirz M., Pichler H., Schwab H. (2014). Protein expression in Pichia pastoris: Recent achievements and perspectives for heterologous protein production. Appl. Microbiol. Biotechnol..

[124] Cregg J.M., Cereghino J.L., Shi J., Higgins D.R. (2000). Recombinant Protein Expression in Pichia pastoris. Mol. Biotechnol..

[125] Daly R., Hearn M.T.W. (2005). Expression of heterologous proteins in Pichia pastoris: A useful experimental tool in protein engineenring and production. J. Mol. Recognit..

[126] Hong S.-Y., Sohn Y.-D., Chung K.-H., Kim D.-S. (2002). Structural and functional significance of disulfide bonds in saxatilin, a 7.7 kDa disintegrin. Biochem. Biophys. Res. Commun..

[127] Hong S.Y., Lee H., You W.K., Chung K.H., Kim D.S., Song K. (2003). The snake venom disintegrin salmosin induces apoptosis by disassembly of focal adhesions in bovine capillary endothelial cells. Biochem. Biophys. Res. Commun..

[128] Higuchi D.A., Almeida M.C., Barros C.C., Sanchez E.F., Pesquero P.R., Lang E.A.S., Samaan M., Araujo R.C., Pesquero J.B., Pesquero J.L. (2011). Leucurogin, a new recombinant disintegrin cloned from Bothrops leucurus (white-tailed-jararaca) with potent activity upon platelet aggregation and tumor growth. Toxicon.

[129] Singhamatr P., Rojnuckarin P. (2007). Molecular cloning of albolatin, a novel snake venom metalloprotease from green pit viper (Trimeresurus albolabris), and expression of its disintegrin domain. Toxicon.

[130] Lin Y.-T.T., Tang C.-H.H., Chuang W.-J.J., Wang S.-M.M., Huang T.-F.F., Fu W.-M.M. (2005). Inhibition of adipogenesis by RGD-dependent disintegrin. Biochem. Pharmacol..

[131] Dennis M.S., Carter P., Lazarus R.A. (1993). Binding Interactions of Kistrin With Platelet Glycoprotein IIb-IIIa: Analysus by Site-Directed Mutagenesis. Proteins Struct. Funct. Bioinform..

[132] Hsu C.-C., Chuang W.-J., Chung C.-H., Chang C.-H., Peng H.-C., Huang T.-F. (2016). Snake Venom Disintegrin Inhibits the Activation of Toll-Like Receptors and Alleviates Sepsis through Integrin alphaVbeta3 Blockade. Sci. Rep..

[133] Chang Y.-T., Shiu J., Huang C., Chen Y.-C., Chen C., Chang Y., Chuang W. (2017). Effects of the RGD loop and C-terminus of rhodostomin on regulating integrin αIIbβ3 recognition. PLoS ONE.

[134] Sohn Y.D., Hong S.Y., Cho K.S., Choi W.S., Song S.W., Bae J.S., Kim D.S., Chung K.H. (2008). Acute and repeated dose toxicity studies of recombinant saxatilin, a disintegrin from the Korean snake (Gloydius saxatilis). Toxicon.

[135] Sohn Y.D., Cho K.S., Sun S.A., Sung H.J., Kwak K.W., Hong S.Y., Kim D.S., Chung K.H. (2008). Suppressive effect and mechanism of saxatilin, a disintegrin from Korean snake (Gloydius saxatilis), in vascular smooth muscle cells. Toxicon.

[136] Kwon I., Hong S.Y., Kim Y.D., Nam H.S., Kang S., Yang S.H., Heo J.H. (2013). Thrombolytic effects of the snake venom disintegrin saxatilin determined by novel assessment methods: A FeCl3-induced thrombosis model in mice. PLoS ONE.

[137] Setlow J.K., Setlow J.K. (2002). Genetic Engineering.

[138] Chen C.-Y., Shiu J.-H., Hsieh Y.-H., Liu Y.-C., Chen Y.-C., Jeng W.-Y., Tang M.-J., Lo S.J., Chuang W.-J. (2009). Effect of D to E mutation of the RGD motif in rhodostomin on its activity, structure, and dynamics: Importance of the interactions between the D residue and integrin. Proteins.

[139] Shiu J.-H., Chen C.-Y., Chen Y.-C., Chang Y.-T., Huang C.-H., Chuang W.-J. (2012). Effect of P to A mutation of the N-terminal residue adjacent to the Rgd motif on rhodostomin: Importance of dynamics in integrin recognition. PLoS ONE.

[140] Gutierrez D.A., Aranda A.S., Carrillo D.A.R., Koshlaychuk M.A., Sanchez E.E., Lucena S.E., Soto J.G., Marceau K., Ruttle P.L., Shirtcliff E.A. (2015). Functional analysis of four single (RGDWL, RGDWM, RGDWP, RGDMN) and two double (RGDNM, RGDMP) mutants: The importance of methionine (M) in the functional potency of recombinant mojastin (r-Moj). Toxicon.

[141] Mezö G., Hudecz F. (2005). Synthesis of Linear, Branched, and Cyclic Peptide Chimera. Peptide Synthesis and Applications.

[142] Jing J., Lu S. (2005). Inhibition of Platelet Aggregation of a Mutant Proinsulin Chimera Engineered by Introduction of a Native Lys-Gly-Asp-containing Sequence. Biotechnol. Lett..

[143] Sánchez E.E., Galán J.A., Russell W.K., Soto J.G., Russell D.H., Pérez J.C. (2006). Isolation and characterization of two disintegrins inhibiting ADP-induced human platelet aggregation from the venom of Crotalus scutulatus scutulatus (Mohave Rattlesnake). Toxicol. Appl. Pharmacol..

[144] Clarke E.A., Trikha M., Markland M.S., Bugge J.S. (1994). Structurally distinct disintegrins contortrostatin and multiquamatin differentially regulate platelet tyrosine phosphorylation. J. Biol. Chem..

[145] Scarborough R.M., Naughton M.A., Teng W., Rose J.W., Phillips D.R., Nannizzi L., Arfsten A., Campbell A.M., Charo I.F. (1993). Design of potent and specific integrin antagonists: Peptide antagonists with high specificity for glycoprotein IIb-IIIa. J. Biol. Chem..

[146] Lynch J.J., Cook J.J., Sitko G.R., Holahan M.A., Ramjit D.R., Mellott M.J., Stranieri M.T., Stabilito I.I., Zhang G., Lynch R.J. (1995). Nonpeptide glycoprotein IIb/IIIa inhibitors. 5. Antithrombotic effects of MK-0383. J. Pharmacol. Exp. Ther..

[147] Lin E., Wang Q., Swenson S., Jadvar H., Groshen S., Ye W., Markland F.S., Pinski J. (2010). The disintegrin contortrostatin in combination with docetaxel is a potent inhibitor of prostate cancer in vitro and in vivo. Prostate.

[148] Ritter M.R., Zhou Q., Markland F.S. (2001). Contortrostatin, a Homodimeric Disintegrin, Actively Disrupts Focal Adhesion and Cytoskeletal Structure and Inhibits Cell Motility Through a Novel Mechanism. Cell Commun. Adhes..

[149] Zhou Q., Sherwin R.P., Parrish C., Richters V., Groshen S.G., Tsao-Wei D., Markland F.S. (2000). Contortrostatin, a dimeric disintegrin from Agkistrodon contortrix contortrix, inhibits breast cancer progression. Breast Cancer Res. Treat..

[150] Swenson S., Ramu S., Markland F.S. (2007). Anti-angiogenesis and RGD-containing snake venom disintegrins. Curr. Pharm. Des..

[151] Marcinkiewicz C., Weinreb P.H., Calvete J.J., Kisiel D.G., Mousa S.A., Tuszynski G.P., Lobb R.R. (2003). Obtustatin: A potent selective inhibitor of alpha1beta1 integrin in vitro and angiogenesis in vivo. Cancer Res..

[152] Staniszewska I., Walsh E.M., Rothman V.L., Gaathon A., Tuszynski G.P., Calvete J.J., Lazarovici P., Marcinkiewicz C. (2009). Effect of VP12 and viperistatin on inhibition of collagen receptors-dependent melanoma metastasis. Cancer Biol. Ther..

[153] Momic T., Katzehendler J., Benny O., Lahiani A., Cohen G., Noy E., Senderowitz H., Eble J.A., Marcinkiewicz C., Lazarovici P. (2014). Vimocin and Vidapin, cyclic KTS peptides, dual antagonists of α1β1/α2β1 integrins with antiangiogenic activity. J. Pharmacol. Exp. Ther..

[154] Lazarovici P., Momic T., Katzehendler J., Shai E., Noy E., Senderowitz H., Eble J.A., Marcinkiewicz C., Varon D. (2015). Vipegitide: A folded peptidomimetic partial antagonist of α2β1 integrin with antiplatelet aggregation activity. Drug Des. Devel. Ther..

[155] Coelho A.L.J., De Freitas M.S., Mariano-Oliveira A., Oliveira-Carvalho A.L., Zingali R.B., Barja-Fidalgo C. (2001). Interaction of disintegrins with human neutrophils induces cytoskeleton reorganization, focal adhesion kinase activation, and extracellular-regulated kinase-2 nuclear translocation, interfering with the chemotactic function. FASEB J..

[156] Coelho A.L.J., De Freitas M.S., Mariano-Oliveira A., Rapozo D.C.M., Pinto L.F.R., Niewiarowski S., Zingali R.B., Marcinkiewicz C., Barja-Fidalgo C. (2004). RGD- and MLD-disintegrins, jarastatin and EC3, activate integrin-mediated signaling modulating the human neutrophils chemotaxis, apoptosis and IL-8 gene expression. Exp. Cell Res..

[157] Neto E.H., Coelho A.L.J., Sampaio A.L.F., Henriques M.d.G.M.O., Marcinkiewicz C., De Freitas M.S., Barja-Fidalgo C. (2007). Activation of human T lymphocytes via integrin signaling induced by RGD-disintegrins. Biochim. Biophys. Acta.

[158] Saldanha-Gama R.F., Moraes J.A., Mariano-Oliveira A., Coelho A.L., Walsh E.M., Marcinkiewicz C., Barja-Fidalgo C. (2010). alpha(9)beta(1) integrin engagement inhibits neutrophil spontaneous apoptosis: Involvement of Bcl-2 family members. Biochim. Biophys. Acta.

[159] Moraes J.A., Frony A.C., Dias A.M., Renovato-Martins M., Rodrigues G., Marcinkiewicz C., Assreuy J., Barja-Fidalgo C. (2015). Alpha1beta1 and integrin-linked kinase interact and modulate angiotensin II effects in vascular smooth muscle cells. Atherosclerosis.

[160] Moraes J.A., Frony A.C., Dias A.M., Renovato-Martins M., Rodrigues G., Marcinkiewicz C., Assreuy J., Barja-Fidalgo C. (2016). Data in support of alpha1beta1 and integrin-linked kinase interact and modulate angiotensin II effects in vascular smooth muscle cells. Data Br..

[161] Hsu C.C., Chuang W.J., Chang C.H., Tseng Y.L., Peng H.C., Huang T.F. (2011). Improvements in endotoxemic syndromes using a disintegrin, rhodostomin, through integrin αvβ3-dependent pathway. J. Thromb. Haemost..

[162] Kim S.I., Kim K.S., Kim H.S., Kim D.S., Jang Y., Chung K.H., Park Y.S. (2003). Inhibitory effect of the salmosin gene transferred by cationic liposomes on the progression of B16BL6 tumors. Cancer Res..

[163] Swenson S., Costa F., Minea R., Sherwin R.P., Ernst W., Fujii G., Yang D., Markland F.S. (2004). Intravenous liposomal delivery of the snake venom disintegrin contortrostatin limits breast cancer progression. Mol. Cancer Ther..

[164] Janib S.M., Gustafson J.A., Minea R.O., Swenson S.D., Liu S., Pastuszka M.K., Lock L.L., Cui H., Markland F.S., Conti P.S. (2014). Multimeric disintegrin protein polymer fusions that target tumor vasculature. Biomacromolecules.

